# Response to Pfirrmann et al.’s comment on *How should we interpret conclusions of TKI-stopping studies*

**DOI:** 10.1038/s41375-023-02128-z

**Published:** 2023-12-27

**Authors:** Junren Chen, Robert Peter Gale

**Affiliations:** 1 National Key Laboratory of Blood Science, National Clinical Research Center for Blood Diseases, Haihe Laboratory of Cell Ecosystem, Institute of Hematology & Blood Diseases Hospital, Chinese Academy of Medical Sciences & Peking Union Medical College, Tianjin, China; 2 Tianjin Institutes of Health Science, Tianjin, China; 3 Centre for Haematology, Department of Immunology and Inflammation, Imperial College of Science, Technology and Medicine, London, UK

**Keywords:** Chronic myeloid leukaemia, Biostatistics

## To the Editor:

We thank Prof. Pfirrmann and colleagues for their *Correspondence* [1]. Our reply:

*Enthusiasm* does not mitigate the potential problem of *survivorship bias*. The likelihood of therapy-free remission (TFR) reflects the biology of someone’s leukaemia and efficacy of tyrosine kinase inhibitor (TKI)-therapy. For every person *A* eligible to enroll in the EURO-SKI trial there was a person *B* who might never achieve deep molecular response (DMR) regardless of the duration of TKI-therapy [1, 2]. Likewise, for every person *C* able to maintain DMR for a prolonged interval and then achieve TFR there was another person *D* who could never achieve any of these endpoints because he/she had a DMR which was subsequently lost.

Considering persons *A*, *B*, *C*, *D,* we conclude the EURO-SKI trial does not provide guidelines for optimizing the duration of anyone’s TKI-therapy or DMR. The authours made no such claim. Because no one like person *B* enrolled in EURO-SKI (inclusion criteria) EURO-SKI is silent on *B*. In contrast, not every eligible person *A* was enrolled in the EURO-SKI trial. Even if trial enrollment was unbiased we have no method to predict whether person *A* would turn out to be person *C* or *D* if TKI-therapy were continued. Moreover, the EURO-SKI data do not prove person *C* would necessarily fail to achieve TFR if TKI-therapy were discontinued earlier. In contrast, person *D* could not prolong his/her duration of DMR because DMR was already lost. Given these considerations to which persons with chronic myeloid leukaemia should we propose prolonging TKI-therapy to improve his/her likelihood of achieving TFR?

We do not contest having a longer interval of DMR is associated with a greater likelihood of achieving TFR after stopping TKI-therapy. The EURO-SKI data support this conclusion. Rather, we challenge the interpretation of the study results by some physicians who conclude trying to prolong DMR will necessarily result in an increased likelihood of achieving TFR. ‘Attempting to maintain a longer duration of DMR’ is a forward-looking *clinical decision* whereas ‘being able to prolong DMR’ is a potential *endpoint* for assessing therapy efficacy. Physicians need to decide on the former whilst the EURO-SKI study addressed the latter. It is important not to confuse these. Whilst we applaud enthusiasm it is not a metric for a valid scientific conclusion. Many scientists have been enthusiastic about ideas later proved wrong. Aristotle was convinced the Earth was the centre of the universe. (He got lots of other things right).

The correct interpretation of Pfirrmann and colleagues’ logistic regression analyses is that ‘being able to achieve a longer duration of DMR’ is a favorable endpoint *after the fact*. It would be much more informative were there a model which could predict which persons can achieve a longer duration of DMR if TKI-therapy were prolonged. There is no such model. Also, even if there were such a model it is not a foregone conclusion such persons (‘person *C*’*)* could only achieve TFR after prolonged TKI-therapy as we described above.

In their article the authours stated: … longer treatment duration … and longer deep molecular response durations … were *associated* with increasing probability of MMR (major molecular response) maintenance at 6 months [2]. Correct. But association is not *cause-and-effect*. The EURO-SKI trial does not inform us of the *best* duration of imatinib-therapy nor whether its conclusions apply to persons receiving other TKIs.

Our *Perspective* focused not on the conduct of the EURO-SKI study the authours of which are to be congratulated but on how readers might mis-interpret the study results and prolong TKI-therapy inappropriately [3].
